# Characteristics of hemolytic activity induced by the aqueous extract of the Mexican fire coral *Millepora complanata*

**DOI:** 10.1186/1678-9199-20-49

**Published:** 2014-11-12

**Authors:** Alejandro García-Arredondo, Luis J Murillo-Esquivel, Alejandra Rojas, Judith Sanchez-Rodriguez

**Affiliations:** Laboratory of Chemical and Pharmacological Natural Products Research, School of Chemistry, Autonomous University of Querétaro, Querétaro, 76010 Mexico; Reef System Unit, Puerto Morelos, Institute of Marine Sciences and Limnology, National Autonomous University of Mexico, Puerto Morelos, Quintana Roo 77500 Mexico

**Keywords:** Cytolysins, Hemolysins, Fire coral, *Millepora complanata*

## Abstract

**Background:**

*Millepora complanata* is a plate-like fire coral common throughout the Caribbean. Contact with this species usually provokes burning pain, erythema and urticariform lesions. Our previous study suggested that the aqueous extract of *M. complanata* contains non-protein hemolysins that are soluble in water and ethanol. In general, the local damage induced by cnidarian venoms has been associated with hemolysins. The characterization of the effects of these components is important for the understanding of the defense mechanisms of fire corals. In addition, this information could lead to better care for victims of envenomation accidents.

**Methods:**

An ethanolic extract from the lyophilized aqueous extract was prepared and its hemolytic activity was compared with the hemolysis induced by the denatured aqueous extract. Based on the finding that ethanol failed to induce nematocyst discharge, ethanolic extracts were prepared from artificially bleached and normal *M. complanata* fragments and their hemolytic activity was tested in order to obtain information about the source of the heat-stable hemolysins.

**Results:**

Rodent erythrocytes were more susceptible to the aqueous extract than chicken and human erythrocytes. Hemolytic activity started at ten minutes of incubation and was relatively stable within the range of 28-50°C. When the aqueous extract was preincubated at temperatures over 60°C, hemolytic activity was significantly reduced. The denatured extract induced a slow hemolytic activity (HU_50_ = 1,050.00 ± 45.85 μg/mL), detectable four hours after incubation, which was similar to that induced by the ethanolic extract prepared from the aqueous extract (HU_50_ = 1,167.00 ± 54.95 μg/mL). No significant differences were observed between hemolysis induced by ethanolic extracts from bleached and normal fragments, although both activities were more potent than hemolysis induced by the denatured extract.

**Conclusions:**

The results showed that the aqueous extract of *M. complanata* possesses one or more powerful heat-labile hemolytic proteins that are slightly more resistant to temperature than jellyfish venoms. This extract also contains slow thermostable hemolysins highly soluble in ethanol that are probably derived from the body tissues of the hydrozoan.

## Background

The genus *Millepora* comprise calcareous cnidarians that are found on coral reefs worldwide, except on the Hawaiian coast, and constitute one of the most important contributors to reef structures [1]. The nematocysts of these hydrozoans can penetrate the human skin and it is well known that contact with them results in burning pain followed by local erythema, urticariform lesions, and pruritus with regression over a 1- to 2-week period [1–4]. Because of their significant toxicity, *Millepora* species are commonly known as “fire corals”. However, although superficially they resemble scleractinian stony corals, they are not true corals. Based on morphological and biological characteristics, *Millepora* species are classified as members of the class Hydrozoa, and not of the class Anthozoa to wich scleractinian corals belong [1]. In addition, *Millepora* species are capable of inducing systemic toxicity in humans, which includes nausea, vomiting, respiratory difficulty, fever, and even nephritic syndrome, acute renal failure, and pulmonary edema [3, 4].

Several toxinological studies have shown that the venom contained in the nematocysts of *Millepora* species is mainly composed of thermolabile protein toxins that display lethal, hemolytic, dermonecrotic, and antigenic properties [5–9]. Conversely, we have found evidence of the presence of important non-protein bioactive compounds in the aqueous extract of *M. complanata*, a plate-like fire coral commonly found along the Mexican Caribbean coast. Previously, we observed that the aqueous extract of this hydrozoan provoked contractile effects on isolated guinea pig ileum segments and rat aortic rings, and also induced hemolysis of rat erythrocytes [10, 11]. By means of a chromatographic analysis of this extract, we detected the presence of a hemolytic protein of approximately 30 kDa and vasoconstrictor proteins of 15, 20, and 61 kDa. In order to evaluate the systemic toxicity of this extract on mice, we previously found that it induced two characteristic types of death depending on the dose administered by intravenous route [12]. Doses equal to or higher than LD_50_ (LD_50_ = 4.62 μg protein/g of body weight) provoked convulsions and death within one minute, whereas doses lower than LD_50_ generally caused death many hours after injection and tissue samples from these animals revealed histological damage to the kidneys and lungs. Denaturation of the proteins contained in the extract, by incubation in a boiling bath for 30 minutes, resulted in the deactivation of their capacity to induce delayed death and histological damage. Surprisingly, the denaturation of the extract did not affect its capacity to induce convulsions and rapid death. Through a three-step chromatographic fractionation, we found a non-protein fraction, called MC1-IIA, which induces vasoconstriction, delayed hemolysis (incubation for four hours at 37°C), and lethal effects on mice. A subsequent analysis showed that this fraction contains polyoxygenated alkylbenzenes that are highly soluble in water and ethanol [12].

The hemolytic activities of several cnidarian venoms are well documented [13]. The importance or implications of *in vivo* erythrocyte lysis by venom hemolysins is not completely understood, but these venom hemolysins are not specific and may affect other cell types [14]. In fact, local damage induced by cnidarians, such as inflammation and dermonecrosis, is attributed to hemolysins injected by the nematocysts [13, 15, 16]. For this reason, it is important to study the hemolytic properties of cnidarian venoms, which could lead to better care for the victims. For example, first aid treatment of accidental contact with jellyfish includes rinsing the site of the sting with some chemical substances. It was found that lidocaine, ethanol, and diluted acetic acid are highly effective in reducing *in situ* nematocyst discharge [17–20]. In addition, it is proposed that thermal treatment (immersion of the sting site in 45°C water or the application of ice packs) may aid in one or two ways: via the deactivation of heat labile proteins in the venom, or via modulation of pain receptors [17, 21]. However, it could present variations in the sensitivity of cnidarian hemolysins to temperature.

At present, the most common recommendations for treatment of injuries caused by contact with fire corals are the same as those for stings provoked by jellyfish. Although information about the hemolytic properties of these hydrozoans is scarce, it is important for the understanding of the defense mechanisms of these organisms. The aim of the present study was to obtain basic data on the hemolytic activity of the aqueous extract of *M. complanata*, not only by investigating the hemolytic properties of the protein compounds, but also by analyzing the hemolysis induced by the non-protein compounds.

## Methods

### Materials

D-glucose, NaCl, MgCl_2_, CaCl_2_, and ethanol absolute (HPLC grade) were purchased from J.T. Baker (USA). Citric acid and sodium citrate were obtained from Sigma (USA). The reagents used in the determination of protein concentration were obtained from Bio-Rad (USA).

### Sample collection and aqueous extract preparation

Fragments of *M. complanata* were collected from the coast of Puerto Morelos, Quintana Roo, Mexico, at a site known as “La Bocana Chica”, in December 2012. The fragments were kept wet with sea water for their transportation to the Reef Systems Academic Unit (in Spanish, Unidad Académica de Sistemas Arrecifales) and then were frozen and stored at –70°C. Then, all fragments were transported in dry ice to our laboratory in Querétaro, Mexico, where extraction was performed.

Nematocyst discharge was induced by stirring the calcareous fragments in deionized water at 4°C for 24 hours. The extract obtained was centrifuged at 3,000 rpm (2,060 × *g*) for 15 minutes at 4°C. This procedure was repeated twice, and the supernatant was freeze-dried and stored at –70°C. The lyophilized product was dissolved in deionized water at a concentration of 150 mg/mL and centrifuged at 3,000 rpm (2,060 × *g*) for 15 minutes at 4°C. Then, the supernatant was filtered through a 0.45 μm pore filter (Millipore, Germany). The filtered solution was stored at –20°C and used to determine the biological effects.

### Protein concentration determination

Protein was determined by the Bradford assay [22], using a standard curve prepared with lyophilized bovine serum albumin.

### Hemolytic activity test

The hemolytic activity was monitored as described for the jellyfish venom *Cassiopea xamachana* [23]. Briefly, for each experiment an aliquot of blood was washed three times with Alsever’s solution composed of 120 mM D-glucose, 30 mM sodium citrate, 7 mM NaCl, and 2 mM citric acid, pH 7.4. Washing was done by low-speed centrifugation (1,000 × *g*, four minutes) of blood at 4°C (Hermle Z 323 K centrifuge, Lab-Tech Instrumentation, Germany). Washed erythrocytes were diluted in 1% Alsever’s solution (v/v) and combined with different amounts of the aqueous extract (0.03, 0.10, 0.32, 1.00, 3.16, 10.00, 31.60, 100.00, and 316.00 μg protein/mL). Then the samples were incubated at 37°C for 30 minutes (Eppendorf AG 22331Thermomixer, Brinkmann Instruments, Germany). Subsequently, the reaction was stopped by centrifuging for four minutes at 1,000 × *g*. The A_415_ of the supernatant fluid containing the hemoglobin released from lysed erythrocytes was measured in a spectrophotometer (Lambda Bio, Perkin Elmer Co., USA). Each experiment was normalized with respect to complete hemolysis, which was measured by diluting the erythrocyte sample in deionized water instead of Alsever’s solution. One hemolytic unit (HU_50_) was defined as the amount of protein sample required to cause 50% hemolysis.

### Hemolytic properties of the aqueous extract

The hemolytic activity of the aqueous extract of *M. complanata* was tested by using blood samples from humans (young males, weighing approximately 70 kg), chicken (males, weighing 600 g), Harley guinea pigs (males, weighing 450 g), Wistar rats (males, weighing 250 g) and CD1 mice (males, weighing 30 g). Subsequent experiments were conducted using samples of rat blood. Drug-free human blood samples were supplied by the Unit of Chemical Services, School of Chemistry, UAQ (Unidad de Servicios Químicos, Facultad de Química, UAQ). Animal blood samples were supplied by the Animal House of the Institute of Neurobiology, UNAM (Bioterio del Instituto de Neurobiología, UNAM). The hemolytic activity rate of the aqueous extract was tested by using different incubation times (10, 20, 30, and 60 minutes).

In order to evaluate the thermal stability of the hemolytic activity induced by the aqueous extract, it was tested after preincubation at different temperatures (4, 25, 37, 45, 60, and 100°C) for 30 minutes. In addition, the optimum temperature of the aqueous extract was tested by utilizing different incubation temperatures (20, 25, 28, 30, 34, 37, 40, 43, 46, and 50°C) for 30 minutes.

The effects of Ca^++^ and Mg^++^ on the hemolytic activity induced by the aqueous extract were evaluated by adding 5 and 10 mM of CaCl_2_ or MgCl_2_ to Alsever’s solution.

### Analysis of the non-protein hemolysins

In a previous study, we found a non-protein fraction in the aqueous extract of *M. complanata* that was highly soluble in water and ethanol, which induced slow hemolytic activity [12]. Hence, in this study the hemolytic activity of the denatured aqueous extract (induced by preincubation at 100°C for 30 minutes) was tested by incubating erythrocytes (0.10, 0.32, 1.00, 3.16, 10.00, 31.60, 100.00, 316.00, and 1000.00 μg/mL) for four hours at 37°C. The ethanolic extract was prepared by maceration of the lyophilized aqueous extract with ethanol for 48 hours. Then the organic extract was filtered (Whatman filter paper n. 1, General Electric Company, USA) and concentrated to solvent free by evaporation in a rotating evaporator (Büchi, V-850, R-114, B-480, Switzerland) at 40°C.

In order to determine the source of the non-protein cytolysins in *M. complanata* aqueous extract, the ethanolic extracts were prepared in the same way, from artificially bleached and normal hydrozoan fragments. It was found that 70% ethanol failed to induce nematocyst discharge and dramatically impaired chemosensitizer-induced discharge response [19]. It was thus assumed that ethanolic extracts from *M. complanata* fragments contain mainly extra-nematocyst material. In order to test this assumption, *M. complanata* nematocysts were isolated by the method described by Radwan [9] and suspended in ethanol to observe their possible discharge. Bleaching induction was performed according to a method described elsewhere [24]. Briefly, immediately after collection, the calcareous hydrozoan fragments were anchored individually onto modeling clay (Plasticine, Flair Leisure Products plc, UK) on acrylic sheets, divided in two groups, and each group was put into an 80-liter acrylic aquarium tank with filtered flowing seawater at 27°C for five days. Then, the temperature of the tank that contained the experimental group was raised to 33°C until bleaching of the fragments was observed (five days). The temperature of the other tank was maintained at 27°C during this period, and these fragments were used as a normal control fragments. All fragments were subsequently frozen and stored at –70°C.

### Statistical analysis

The program Prism version 5.00 (GraphPad Software, USA) was used for all analysis of data and statistics. Each concentration-response curve was plotted in triplicate using erythrocytes from one animal. For each curve, the HU_50_ was estimated by fitting log(agonist) vs. response using non-linear regression analysis. The HU_50_ of each experimental condition was expressed as mean ± SEM (n = 3) and multiple comparisons were made by one-way analysis of variance followed by Tukey’s test. All tests were considered statistically significant at *p* <0.05.

## Results

### Properties of the hemolytic activity of the aqueous extract

**Figure 1:**
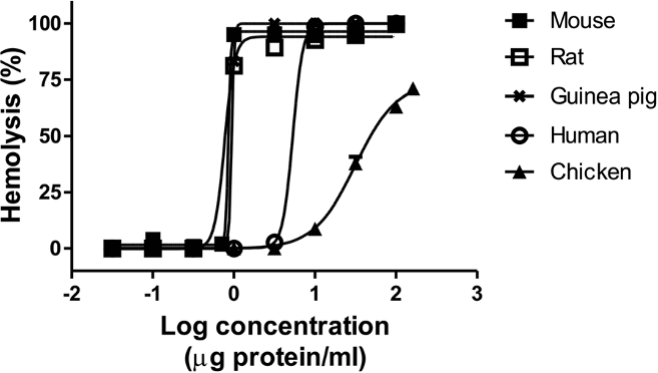
**Representative concentration-response curves of hemolysis induced by** ***M. complanata*** **aqueous extract on erythrocytes of various species.**

*M. complanata* aqueous extract was tested for its hemolytic activity on erythrocytes of various species. These results showed that the extract induces concentration-dependent hemolytic activity in all species used in this study (Figure 1). Comparisons between HU_50_ values did not show differences between guinea pig, rat and mouse. A slight difference, statistically insignificant, was observed among hemolysis induced on human erythrocytes and other red blood cells. Undoubtedly, chicken erythrocytes were less susceptible to lysis, resulting in a hemolytic effect with lower potency and less efficacy. Table 1 shows the HU_50_ and E_max_ values estimated by non-linear regression analysis.

**Table 1:** **Values of hemolytic activity (HU**_**50**_ **and E**_**max**_ **) of the** ***M. complanata*** **aqueous extract on erythrocytes of various species**

Species	HU _50_ (μg protein/mL)	E _max_
Human	5.36 ± 0.88	99.78 ± 0.98
Chicken	24.55 ± 6.93*	80.85 ± 6.82
Guinea pig	0.56 ± 0.01	105.45 ± 2.15
Rat	0.49 ± 0.09	98.87 ± 3.34
Mouse	0.80 ± 0.03	107.15 ± 1.65

*Significantly different from all HU_50_ values (p <0.05).

When the hemolytic activity of the aqueous extract was tested by using different incubation times (10, 20, 30, and 60 minutes), no significant differences were observed (Table 2). These results indicate that the extract induces a rapid hemolytic activity and after ten minutes its potency is similar to that observed after 30 minutes of incubation.

**Table 2:** **HU**_**50**_ **values of the** ***M. complanata*** **aqueous extract obtained by using different incubation times with rat erythrocytes**

Time (min)	HU _50_ (μg protein/mL)
10	0.58 ± 0.11
20	0.52 ± 0.14
30	0.41 ± 0.05
60	0.20 ± 0.03

**Figure 2:**
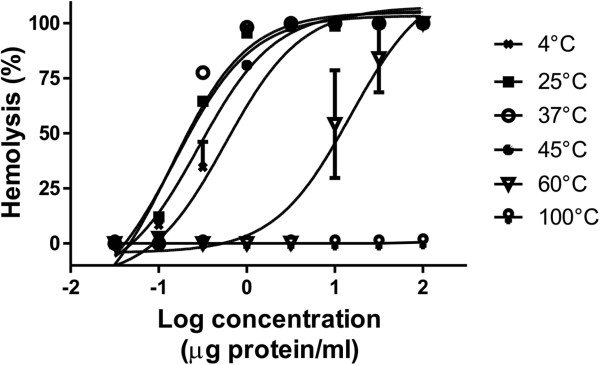
**Representative concentration-response curves of hemolysis induced by** ***M. complanata*** **aqueous extract after preincubation at different temperatures for 30 minutes.**

The hemolytic activity induced by the aqueous extract exhibited thermal instability. Figure 2 shows representative hemolytic concentration-response curves of the extract after preincubation at different temperatures and Table 3 shows the corresponding HU_50_ values. The hemolytic activity of the extract was not significantly affected when it was preincubated at temperatures below 45°C for 30 minutes. The hemolytic potency of the extract was significantly reduced after incubation at 60°C and was destroyed at 100°C. However, when the hemolytic activity of the extract was assayed by using different temperatures of incubation with rat erythrocytes for 30 minutes, no significant differences were observed at temperatures over 28°C (Table 4). However, at temperatures lower than 28°C, hemolytic activity was significantly reduced. Temperatures of incubation above 50°C were not reported since the erythrocytes were lysed as a result of the high temperature.

**Table 3:** **HU**_**50**_ **values of the** ***M. complanata*** **aqueous extract obtained after preincubation at different temperatures for 30 minutes with rat erythrocytes**

Temperature (°C)	HU _50_ (μg protein/mL)
4	0.41 ± 0.12
25	0.35 ± 0.2
37	0.17 ± 0.03
45	0.50 ± 0.07
60	12. 97 ± 2.06*
100	–

*Significantly different from all HU_50_ values (p <0.05).

**Table 4:** **HU**_**50**_ **values of the** ***M. complanata*** **aqueous extract obtained by using different incubation temperatures for 30 minutes with rat erythrocytes**

Temperature (°C)	HU _50_ (μg protein/mL)
20	60.25 ± 3.88*
25	15.06 ± 4.53*
28	7.07 ± 1.45
30	8.69 ± 0.82
34	4.15 ± 0.29
37	2.03 ± 0.35
40	2.06 ± 0.66
43	2.07 ± 0.33
46	2.00 ± 0.15
50	2.25 ± 0.14

*Significantly different from all HU_50_ values (p <0.05).

When the hemolytic activity of the aqueous extract was assayed by using Alsever’s solution with Ca^++^ and Mg^++^ at two concentrations (5 and 10 mM), it was observed that this activity was independent of the presence of these divalent cations. Table 5 shows the HU_50_ obtained in the presence of these ions.

**Table 5:** **HU**_**50**_ **values of the** ***M. complanata*** **aqueous extract obtained by using two different concentrations of Ca** ^**++**^ **and Mg** ^**++**^ **in Alsever’s solution**

	HU _50_ (μg protein/mL)
Control	0.48 ± 0.12
5 mM Ca^++^	0.45 ± 0.04
10 mM Ca^++^	0.35 ± 0.19
5 mM Mg^++^	0.68 ± 0.37
10 mM Mg^++^	0.39 ± 0.19

### Analysis of the non-protein hemolysins

**Figure 3:**
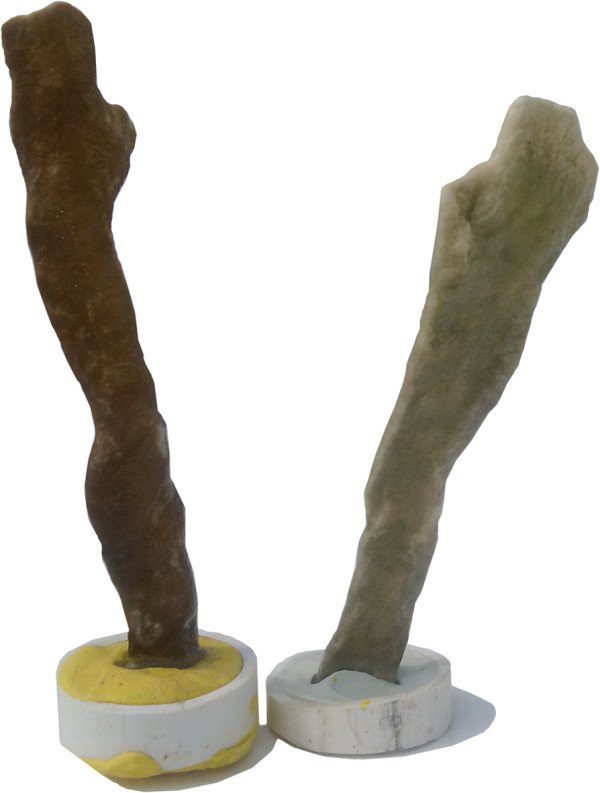
**Photograph of treated** ***M. complanata*** **fragments that were employed to induce bleaching.** A control fragment is shown on the left and a bleached fragment is shown on the right.

**Figure 4:**
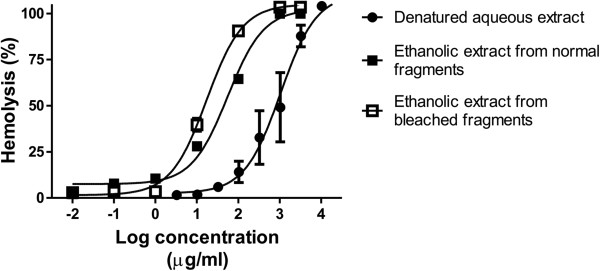
**Representative concentration-response curves of hemolysis induced by non-protein extracts from** ***M. complanata*** **.**

**Figure 5:**
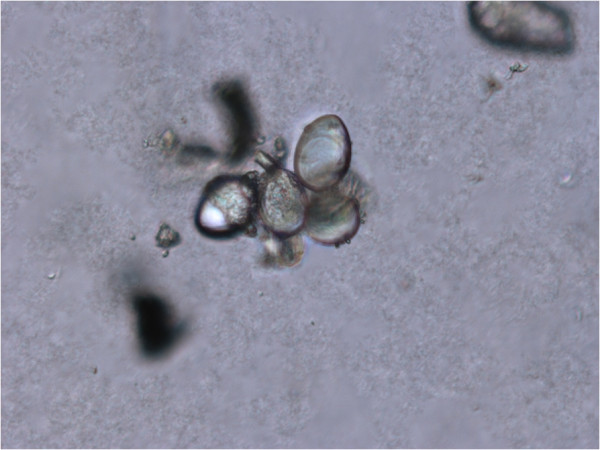
**Light microscopy image showing isolated nematocysts that remained undischarged when suspended in ethanol.** Magnification 400 ×.

To evaluate the hemolytic activity induced by non-protein compounds contained in the aqueous extract of *M. complanata*, the hemolytic test was performed by incubating the denatured extracts with the erythrocytes for four hours at 37°C. As a result, the denatured aqueous extract (protein content of 3.077% w/w) induced a delayed concentration-dependent hemolysis (HU_50_ = 1,050.00 ± 45.85 μg/mL). The ethanolic extract prepared by maceration of the lyophilized aqueous extract (protein content of 0% w/w) induced a hemolytic effect (HU_50_ = 1,167.00 ± 54.95 μg/mL) with a potency similar to that induced by the denatured aqueous extract. Ethanolic extracts, obtained by maceration of bleached and normal fragments (Figure 3), also induced a concentration-dependent hemolytic activity. The hemolysis induced by the ethanolic extract (protein content of 0.699% w/w) from control fragments (HU_50_ = 55.48 ± 14.36 μg/mL) was similar to that induced by the ethanolic extract (protein content of 0.067% w/w) from bleached fragments (HU_50_ = 17.35 ± 2.77 μg/mL). These organic extracts were significantly more potent than the denatured aqueous extract. Figure 4 shows representative concentration-response curves of the hemolysis induced by these extracts. In addition, when isolated nematocysts suspended in ethanol were observed by microscopy, it was found that they were undischarged (Figure 5).

## Discussion

Cnidarian venoms are complex mixtures of natural toxins that vary in activity and composition. To date, research on these venoms has shown that they contain two main types of toxins, namely: neurotoxins and cytolysins [25]. Cnidarian neurotoxins are generally low-molecular-weight peptides and clearly have an aggressive purpose as they induce paralysis in the prey [25, 26]. However, there are also peptide neurotoxins that have a defensive role as they induce intense pain [27]. Cnidarian cytolysins are a diverse group of peptides and proteins that help in the pre-digestion of prey as well as in the dissemination of the venom by inducing local tissue destruction [14, 15]. These toxins attract considerable interest due to their possible application as models for the study of many membrane processes, and due to their possible use in biotherapeutic applications such as the selective killing of parasites and cancer cells [28, 29]. Moreover, the local damage induced by cnidarian venom has been attributed to cytolysins [13, 15, 16]. Therefore, characterization of these cytolysins may lead to better care for the victims of envenomation accidents.

Cnidarian cytolysins induce hemolysis against erythrocytes of many different species [13]. With some exceptions, these cytolysins are contained in stinging capsules called nematocysts that inject their content into prey through a harpoon-like structure after an appropriate stimulus [30]. Some cnidarians possess nematocysts capable of penetrating the human skin and inducing local and systemic symptoms, which comprises a serious threat to human health [17, 20, 31]. In the Mexican Caribbean, *Millepora* species are noted for their ability to induce burning pain and local reactions on human skin after contact. In fact, these species possess penetrating nematocysts [2]. In a previous study, we reported the concentration-dependent hemolytic activity of the aqueous extract of *M. complanata* on rat erythrocytes and found that this activity was heat-labile, which suggested that the hemolysins responsible for this activity were proteins [11]. In the present study, we found that this extract also induced hemolysis on human, chicken, guinea pig and mouse erythrocytes under the same conditions as those of the previous study. These results showed that rodent erythrocytes were more susceptible to lysis induced by the extract than human and chicken erythrocytes, which could be due to differences in the composition of membrane lipids, since several cnidarian cytolysins preferably inserts in membranes that have a specific lipid in a greater proportion [28].

Studies on the hemolytic properties of protein cytolysins from cnidarian venoms, including the *Millepora* species, have shown that they induce rapid hemolysis [23, 32–35]. In this study, hemolytic reaction tests revealed that after incubation with the erythrocytes for ten minutes at 37°C, the aqueous extract of *M. complanata* induces a concentration-dependent hemolysis similar to that previously reported after incubation for 30 minutes. As previously observed, the hemolytic activity of this aqueous extract was temperature-sensitive. Preincubation at 60°C significantly reduced the hemolytic activity of the extract and this activity was almost completely abolished when the extract was preincubated at 100°C for 30 minutes. Loss of hemolytic activity might be due to heat denaturation and presence of protease. However, in a previous study we found that the aqueous extract of this hydrozoan does not exhibit protease activity [12].

Protease activity has been identified in the venoms of other cnidarians such as the jellyfish *Rhopilema esculentum* and *Carybdea alata* [32, 36]. The hemolytic potency of these venoms was greatly reduced after preincubation at 45°C for 30 minutes. Apparently, the aqueous extract of *M. complanata* is slightly more resistant to temperature than jellyfish venoms and this could be due to the absence of proteases. Undoubtedly, the complete abolishment of activity by heating at 100°C is attributed to denaturation of hemolytic proteins. Moreover, when the hemolytic activity of the extract was assayed at different incubation temperatures, no significant differences were observed at temperatures over 28°C, which indicates that hemolysins of the extract are relatively stable in a range of temperatures from 28 to 50°C. At temperatures lower than 28°C, the hemolytic activity of the aqueous extract was significantly reduced, which shows that warm temperatures stimulate the hemolytic reaction. Hemolysins from jellyfish venoms usually have their optimum activity at temperatures below 45°C. For example, the optimum temperature for incubation of *Stomolophus meleagris* venom was 37°C relatively to its hemolytic activity [37]. The heat sensitivity of jellyfish cytolysins is one of the reasons for applying hot water (42-45°C) to sting sites [17, 21]. However, it should be considered that this first aid might not be the most suitable care to inactivate the proteins that induce local reactions caused by contact with *M. complanata*.

To date, the hemolytic mechanism of *Millepora* venoms has not yet been defined. Despite the variability in the composition of cnidarian venoms, there is evidence that supports the idea that actinoporin-like toxins and phospholipase A_2_ (PLA_2_) toxins are the basic components of the cnidarian venom system [38]. Some studies have shown that the hemolytic activity induced by *Millepora* venoms might be associated with the presence of PLA_2_ proteins. For instance, it was reported that *Millepora* species present higher PLA_2_ activity levels than other cnidarians [39]. Shiomi *et al.* [35] reported that the venoms of *M. dichotoma* and *M. platyphylla* exhibit PLA_2_ activity, but other enzymatic activity such as hyaluronidase and protease were not detected. Radwan and Aboul-Dahab [40] isolated a 32.5 kDa PLA_2_ protein, named milleporin-1, in the venom of *M. platyphylla* that showed a significant contribution to the overall hemolysis of human erythrocytes. As concerns *M. complanata*, we found in a previous study that the aqueous extract of this species presents PLA_2_ activity, and its hemolytic activity was significantly decreased after incubation with the PLA_2_ inhibitor *p*-bromophenacyl bromide. In a preliminary fractionation by HPLC of this extract we detected a hemolysin with a relative molecular weight similar to that of milleporin-1 [11].

The hemolytic activity of PLA_2_ proteins may be due to their enzymatic activity resulting in hydrolysis of cellular membrane phospholipids, or indirectly through the generation of toxic free fatty acids and lysophospholipids [41]. A common characteristic of most secreted PLA_2_ proteins is that the presence of a millimolar concentration of Ca^++^ is required for catalytic activity. In this study, we observed that the hemolytic activity of the aqueous extract of *M. complanata* was independent of the presence of Ca^2+^ and Mg^2+^. The effect of these cations on the hemolytic activity of cnidarian venoms is variable, which may be due to structural differences that contribute to their cytolytic activity [28, 32, 33, 37, 42]. It is known that PLA_2_ from venoms induce other toxic effects that are either dependent or independent on catalytic activity, such as neurotoxicity, cardiotoxicity, myotoxicity or even digestive activity [15, 41].

An interesting feature of the aqueous extract of *M. complanata* is that it contains non-protein cytolysins that induce slow hemolytic activity. The hemolytic activity of this extract was evaluated after denaturation of its protein content and we found that after four hours of incubation with erythrocytes concentration-dependent hemolysis had been induced. Based on the previous detection of a hemolytic non-protein fraction that was highly soluble in water and ethanol [12], we also evaluated the hemolytic activity of an ethanolic extract from the lyophilized aqueous extract and observed a very similar hemolytic activity with that of denatured aqueous extract. Protein content was not detectable in this ethanolic extract, which suggested that these non-protein slow hemolysins were completely extracted by ethanol from the aqueous extract.

The presence of non-protein toxins in other cnidarians is common [13, 25]. The most interesting example is palytoxin, a complex polyether compound that was first isolated with ethanol from the zoanthid *Palythoa toxica* (class Anthozoa). This hemolysin, although potent, acts slowly on erythrocytes from several species and induces cytotoxic activity on cultured cells [43]. In order to determine the source of the non-protein hemolysins of the aqueous extract of *M. complanata*, we prepared ethanolic extracts from bleached (without symbiotic zooxanthellae) and control fragments of the hydrozoan. These extracts were prepared based on a study that showed that 70% ethanol failed to induce nematocyst discharge and dramatically impaired chemosensitizer-induced discharge response [19]. When isolated, nematocysts from *M. complanata* that were suspended in ethanol were observed by microscopy, and it was found that they were undischarged. In consideration of this fact, the extra-nematocyst material may be extracted with ethanol from the fragments of the hydrozoan.

The results of the evaluation of these ethanolic extracts showed that the extract from bleached fragments was slightly more potent than the extract from control fragments, while both were much more potent than the denatured aqueous extract. The reason for the higher potency of these extracts is that they present these thermostable slow hemolysins in a higher concentration than the denatured aqueous extract. These results indicate that the source of these non-protein hemolysins is the extra-nematocyst tissue of *M. complanata* and not the nematocysts or the symbiotic zooxanthellae. The presence of bioactive secondary metabolites in the body tissue of hydrozoans is very common and it is believed that these organisms are protected from predation not only by their nematocysts but also by their non-nematocystic toxins, such as secondary metabolites and pore forming proteins [15]. Therefore, further research on the toxins contained in the aqueous extract of *M. complanata* is essential to determine their identity and mechanisms of action, and to establish similarities with other hydrozoans and differences with other cnidarians.

## Conclusions

The present results show that the *M. complanata* aqueous extract possesses one or more powerful heat-labile hemolytic proteins that are slightly more resistant to temperature than jellyfish venoms. These hemolysins do not require a long incubation time and are Ca^++^ and Mg^++^ independent. This aqueous extract also contains slow thermostable hemolysins that are highly soluble in ethanol. We inferred that these possible cytolytic secondary metabolites are derived from the body tissues of the hydrozoan. This hypothesis requires further research in future studies.

### Ethics committee approval

The present study was conducted according to and approval of the National Commission of Aquaculture and Fishing, the Secretary and Agriculture, Livestock, Rural Development, Fishing and Feeding of Mexican Federal Government (permission number PFP/DGOPA-071/13).
